# Diosmin Administration Slightly Counteracted the Changes in Bone Mechanical Properties Induced by Experimental Type 1 Diabetes in Rats

**DOI:** 10.3390/ph18050715

**Published:** 2025-05-13

**Authors:** Kacper Grzywnowicz, Piotr Londzin, Sylwia Klasik-Ciszewska, Weronika Borymska, Maria Zych, Ilona Kaczmarczyk-Żebrowska, Joanna Folwarczna

**Affiliations:** 1 Department of Pharmacology, Faculty of Pharmaceutical Sciences in Sosnowiec, Medical University of Silesia, Katowice, 41-200 Sosnowiec, Poland; kacper.grzywnowicz@sum.edu.pl (K.G.); piotr.londzin@vp.pl (P.L.); sklasik-ciszewska@sum.edu.pl (S.K.-C.); 2 Department of Pharmacognosy and Phytochemistry, Faculty of Pharmaceutical Sciences in Sosnowiec, Medical University of Silesia, Katowice, 41-200 Sosnowiec, Poland; weronika.borymska@sum.edu.pl (W.B.); mzych@sum.edu.pl (M.Z.); izebrowska@sum.edu.pl (I.K.-Ż.)

**Keywords:** diosmin, experimental type 1 diabetes, bone, rats

## Abstract

**Background**: There is interest in substances of plant origin that may have health-promoting effects regarding diabetes and its complications, including increased risk of fractures. Diosmin, which exerts, among others, antioxidant, anti-inflammatory and some antidiabetic effects, is widely used in the treatment of chronic venous disease. Since diabetic microvascular complications can contribute to bone damage, the aim of this study was to investigate the effects of diosmin on the skeletal system of rats with experimental streptozotocin-induced type 1 diabetes. **Methods**: The study was conducted on mature male Wistar rats. Diosmin administration (50 and 100 mg/kg/day p.o.) started two weeks after the streptozotocin injection and lasted for four weeks. Serum bone turnover markers, bone mass and mineralization, mechanical properties and histomorphometric parameters were evaluated. **Results**: Diabetes induced strong disorders of bone metabolism and decreases in cancellous and compact bone strength. The administration of diosmin had no beneficial effect on serum bone turnover markers and bone mass and mineralization in diabetic rats. However, at a lower dose, it improved some bone mechanical properties; no effect of diosmin at a higher dose on bone mechanical parameters was demonstrated. **Conclusions**: The results of the present study do not support the use of diosmin in order to counteract the skeletal complications of diabetes.

## 1. Introduction

Skeletal complications may occur in both type 1 (T1D) and type 2 (T2D) diabetes. Patients with T1D often show decreased bone mineral density (BMD) and muscle mass, while patients with T2D show no such changes or may have increased BMD and muscle mass [1,2]. However, both patients with T1D and T2D are at higher risk for fractures, and the risk is greater with T1D [3,4,5,6]. Chronic hyperglycemia causes the formation of advanced glycation end products (AGEs) and reactive oxygen species (ROS), which lead to increased oxidative stress and increased inflammation; these changes are responsible for impaired osteoblast function and reduced bone mass in T1D [7,8,9]. These same factors are involved in the development of microvascular complications in diabetes, including retinopathy, neuropathy and nephropathy [10]; some authors attribute diabetes-induced skeletal damage to microvascular changes, among other factors [4,11,12,13,14].

There is interest in searching for substances of plant origin that could be useful in preventing unfavorable bone changes, including those induced by diabetes. This interest has been encouraged by numerous experimental studies reporting the favorable effects of different polyphenols, including flavonoids, in different models of bone loss (for reviews, see [15,16,17]), as well as many studies indicating the beneficial effects of the same substances of plant origin in diabetes (especially type 2 diabetes; for recent reviews, see [15,18]). However, the effects of the majority of those substances on bone were not confirmed in carefully planned, big enough, randomized clinical trials. A recent review of clinical trials concerning effects of plant-derived medicines concluded that more studies are needed to confirm their efficacy and safety in the management of osteoporosis [19]. Based on previous studies, it can be assumed that some substances exhibiting antidiabetic, antioxidant and anti-inflammatory effects may have beneficial effects on the skeletal system in diabetes. Protective effects on the microcirculation may also be useful. A substance with potential favorable effects on the skeletal system in diabetes may be diosmin.

Diosmin is a flavonoid found in small amounts in several plants, including *Scrophularia nodosa* L. and citrus fruits; it is a glycoside of diosmetin (diosmetin 7-O-rutinoside). Diosmin is closely related to hesperidin, a flavonoid present in large quantities in the peel of citrus fruits. Diosmin is produced from hesperidin, which is extracted from the pericarp of citrus fruits and subsequently dehydrogenated. Diosmin exerts antioxidant and anti-inflammatory effects and benefits the vascular system. Diosmin is widely used as a drug/dietary supplement to treat vascular disorders such as chronic venous disease or hemorrhoidal disease (it is often used with hesperidin) [20,21,22,23]. Diosmin has also been reported to exert other activities, including anticancer, immunomodulatory and antidiabetic effects [24,25].

The beneficial effects of diosmin in diabetes and its complications may be due to its diverse actions, as reviewed in references [20,21,22,24]. Among others, diosmin increased the expression of the GLUT4 transporter in experimental diabetes; the increased expression of this transporter leads to improved glucose uptake [26]. Diosmin was also reported to reduce the expression of pro-inflammatory cytokines in experimental diabetes [27,28]. The antioxidant activity of diosmin was demonstrated in different experimental conditions, including diabetes [28,29,30]. The protective effect of diosmin on blood vessels may be related to an increase in vessel wall tension, improvement in microcirculation, inhibition of inflammation and reduction in capillary permeability [22,30].

Several studies have shown the beneficial effects of diosmin on the skeletal system. To date, such effects have been demonstrated in rats with osteoporosis induced by excess glucocorticoids [31], rats with estrogen deficiency [32] and in bone disorders caused by chronic kidney disease in rats [33]. The administration of diosmin with hesperidin improved bone healing after osteotomy [32]. An in vitro study, confirmed by an in vivo experiment, showed that biocomposite scaffolds containing diosmin may have potential therapeutic applications in bone regeneration [34].

Although studies on the effects of diosmin on osteoporotic or diabetes-induced metabolic disorders are not abundant, diosmin belongs to the most-often prescribed or used in self-medication flavonoids, and as such should be thoroughly examined. The effects of diosmin on skeletal changes induced by the development of diabetes have not yet been studied. Given the potential impact of diosmin in improving vascular health, which may be impaired in diabetes, it can be hypothesized that the use of diosmin may help reduce the adverse effects of diabetes on the skeletal system. The aim of the present study was to investigate the effects of diosmin on the skeletal system of rats with experimental streptozotocin (STZ)-induced T1D.

## 2. Results

### 2.1. Effect of Diosmin on Serum Markers of Bone Turnover in T1D Rats

**Figure 1:**
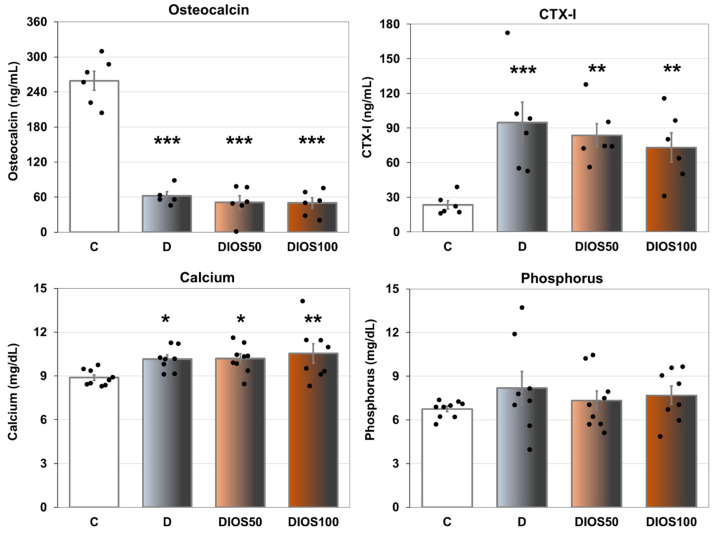
The effects of diosmin administered for 4 weeks on the serum markers of bone turnover in rats with STZ-induced T1D. The results are presented as means ± the standard error of the mean (SEM; *n* = 5–6 for osteocalcin and CTX-I; *n* = 8–9 for calcium and phosphorus). C—healthy control rats (*n* = 9); D—diabetic control rats (*n* = 8); DIOS50—diabetic rats treated with diosmin at a dose of 50 mg/kg p.o. for 4 weeks (*n* = 9); DIOS100—diabetic rats treated with diosmin at a dose of 100 mg/kg p.o. for 4 weeks (*n* = 8). CTX-I—C-terminal telopeptide fragments of type I collagen. Each bullet represents one data point. One-way analysis of variance (ANOVA) followed by Fisher’s LSD post hoc test were used for the evaluation of the significance of the results. The ANOVA results for osteocalcin were as follows: F_3,19_ = 77.260, *p* < 0.001; for CTX-I: F_3,20_ = 6.634, *p* = 0.003; for calcium: F_3,30_ = 3.631, *p* = 0.024; for phosphorus: F_3,30_ = 0.745, *p* = 0.534. Statistical significance in the post hoc test is indicated as follows: * *p* < 0.05, ** *p* < 0.01, *** *p* < 0.001 in comparison with the healthy control rats (C group).

As previously reported [29], administration of diosmin (50 or 100 mg/kg p.o. daily for 4 weeks) had no effect on blood glucose levels in the diabetic rats whose bones were examined in this study. There was no significant effect of the oral administration of diosmin on the serum markers of bone turnover in relation to the diabetic control rats; the bone resorption marker (CTX-I) remained increased and the bone formation marker (osteocalcin) remained decreased compared to the healthy control rats (Figure 1). Also, the serum concentration of calcium, increased due to diabetes, was not affected by diosmin administration. Diabetes and diosmin administration did not affect the serum phosphorus concentration.

Summing up, the administration of diosmin did not alleviate profound, diabetes-induced changes in the serum concentrations of bone turnover markers and calcium.

### 2.2. Effect of Diosmin on Bone Macrometric Parameters, Mass, Density and Mineralization in Femur and Tibia of T1D Rats

Diabetes had a profound effect on most of the studied parameters of the femur and tibia (Table 1 and Supplementary Materials, Table S1). Significant decreases in bone mass, macrometric parameters, bone density, bone mineral mass and bone mineral content, and mass of bone water and bone organic substances were observed compared to the healthy control rats. Similar effects were observed in the L4 vertebra (Supplementary Materials, Table S2). The administration of diosmin to rats with diabetes had no significant effect on the parameters studied regarding bone mass and mineralization in comparison with the diabetic controls.

Summing up, the decrease in bone formation and increase in bone resorption in diabetic rats led to decreases in their bone macrometric parameters, mass and mineralization, which were not counteracted by diosmin administration.

### 2.3. Effect of Diosmin on Mechanical Properties of Proximal Tibial Metaphysis in T1D Rats

**Figure 2:**
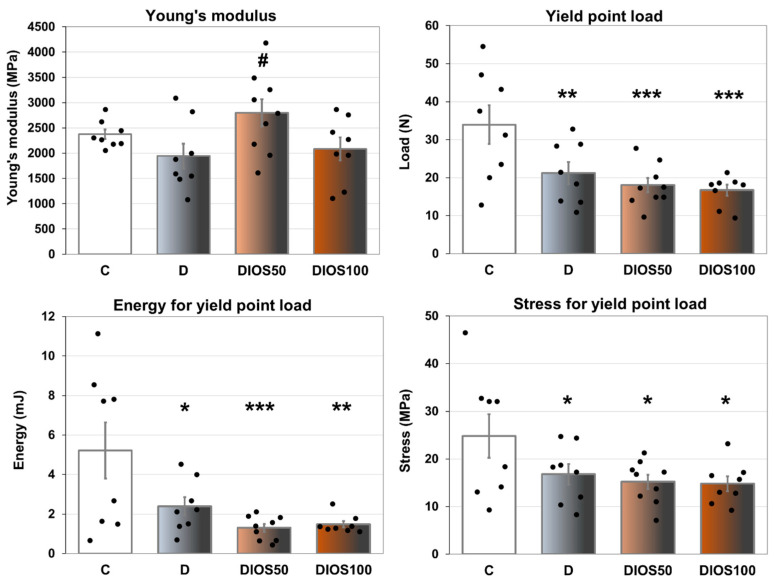
The effects of diosmin administered for 4 weeks on the mechanical properties of the proximal tibial metaphysis in rats with STZ-induced T1D. The results are presented as means ± the standard error of the mean (SEM; *n* = 8–9). C—healthy control rats (*n* = 9); D—diabetic control rats (*n* = 8); DIOS50—diabetic rats treated with diosmin at a dose of 50 mg/kg p.o. for 4 weeks (*n* = 9); DIOS100—diabetic rats treated with diosmin at a dose of 100 mg/kg p.o. for 4 weeks (*n* = 8). Each bullet represents one data point. One-way analysis of variance (ANOVA) followed by Fisher’s LSD post hoc test were used for the evaluation of the significance of the results. The ANOVA results for Young’s modulus are as follows: F_3,29_ = 2.961, *p* = 0.049; for yield point load: F_3,29_ = 6.324, *p* = 0.002; for energy for yield point load: F_3,29_ = 5.984, *p* = 0.003; for stress for yield point load: F_3,29_ = 2.984, *p* = 0.047. Statistical significance in the post hoc test is indicated as follows: * *p* < 0.05, ** *p* < 0.01, *** *p* < 0.001 in comparison with the healthy control rats (C group); # *p* < 0.05 in comparison with the diabetic control rats (D group).

T1D significantly worsened the mechanical properties of the proximal tibial metaphysis (composed mainly of cancellous bone) in rats. Reduced values of load, energy and stress for yield point as well as a non-significant reduction in Young’s modulus were shown in the tibial metaphysis of the diabetic control rats compared to the healthy controls (Figure 2, results for displacement—Table 2). Similar results were obtained for the parameters for maximum load and fracture points (Table 2). The administration of diosmin at a dose of 50 mg/kg p.o. increased Young’s modulus in the proximal tibial metaphysis compared to the diabetic control rats. The administration of diosmin did not affect any other mechanical parameters of cancellous bone of the tibial metaphysis in diabetic rats.

**Figure 3:**
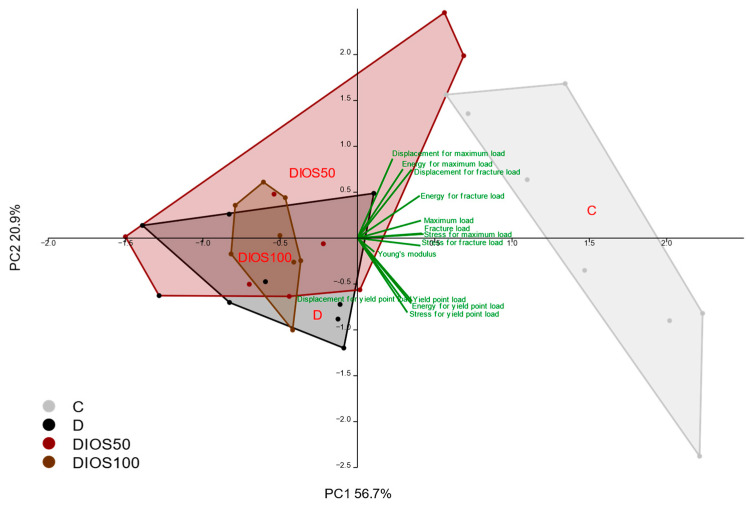
The principal component analysis (PCA) biplot of the results concerning the mechanical properties of the proximal tibial metaphysis in rats with STZ-induced T1D. The green font and green lines indicate the correlations of the measured variables against the experimental groups. The shade of light gray denotes the C group (healthy control rats, *n* = 9); the shade of dark gray denotes the D group (diabetic control rats, *n* = 8); the shade of red denotes the DIOS50 group (diabetic rats treated with diosmin at a dose of 50 mg/kg p.o., *n* = 9); the shade of brown denotes the DIOS100 group (diabetic rats treated with diosmin at a dose of 100 mg/kg p.o., *n* = 8). The names of the groups are marked in red fonts inside the convex hulls comprising individuals from the same group. The statistical significance between the groups along the PCs was analyzed by MANOVA followed by Fisher’s LSD post hoc test. The statistical significance of the MANOVA: *p* < 0.001. The statistical significance for PC1: *p* < 0.001. Statistically significant results of the post hoc comparisons for PC1: C versus D—*p* < 0.001; C versus DIOS50—*p* < 0.001; C versus DIOS100—*p* < 0.001. For PC2, *p* = 0.592.

Principal component analysis (PCA) carried out on the results concerning the mechanical properties of the proximal tibial metaphysis confirmed the strong effect of diabetes, which was not counteracted by diosmin administration (Figure 3).

Summarizing, diabetes-induced changes in bone metabolism led to the profound worsening of the mechanical properties of cancellous bone, which was not counteracted by the administration of diosmin, with the exception of improvement in one parameter—Young’s modulus—after the administration of the lower dose.

### 2.4. Effect of Diosmin on Mechanical Properties of Femoral Diaphysis and Femoral Neck in T1D Rats

**Figure 4:**
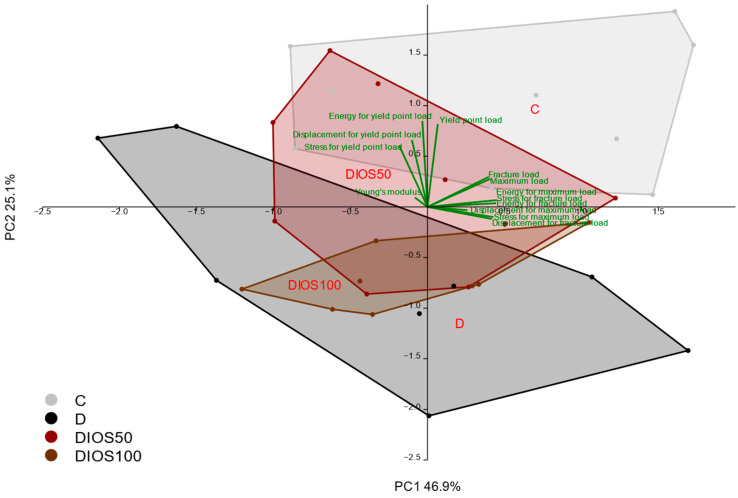
The principal component analysis (PCA) biplot of the results concerning the mechanical properties of the femoral diaphysis in rats with STZ-induced T1D. The green font and green lines indicate the correlations of the measured variables against the experimental groups. The shade of light gray denotes the C group (healthy control rats, *n* = 9); the shade of dark gray denotes the D group (diabetic control rats, *n* = 8); the shade of red denotes the DIOS50 group (diabetic rats treated with diosmin at a dose of 50 mg/kg p.o., *n* = 9); the shade of brown denotes the DIOS100 group (diabetic rats treated with diosmin at a dose of 100 mg/kg p.o., *n* = 8). The names of the groups are marked in red fonts inside the convex hulls comprising individuals from the same group. The statistical significance between the groups along the PCs was analyzed by MANOVA followed by Fisher’s LSD test. The statistical significance of the MANOVA: *p* < 0.001. The statistical significance for PC2: *p* < 0.001. Statistically significant results of the post hoc comparisons for PC2: C versus D—*p* < 0.001; C versus DIOS50—*p* < 0.05; C versus DIOS100—*p* < 0.001; D versus DIOS50—*p* < 0.05. For PC1, *p* = 0.326.

The effects of diabetes on the mechanical properties of compact bone of the femoral diaphysis were much weaker than those in the proximal tibial metaphysis (Table 3). There were only significant reductions in the load and energy for the yield point and the values of the maximum and fracture loads in the diabetic control rats compared to the healthy control rats. The deleterious effects of diabetes on the mechanical properties were confirmed by PCA (Figure 4). There was no significant effect of diabetes on the strength of the femoral neck consisting of cancellous and compact bone. The administration of diosmin at a dose of 50 mg/kg p.o. to diabetic rats increased the values of load and energy for the yield point in the femoral diaphysis relative to the diabetic controls. The administration of diosmin had no effect on other mechanical parameters of compact bone of the femoral diaphysis or on the strength of the femoral neck in diabetic rats. However, PCA confirmed the slight favorable effect of diosmin administered at the lower dose on the mechanical properties of the femoral diaphysis in diabetic rats (Figure 4).

Concluding, the diabetes-induced deterioration of compact bone mechanical properties (which was weaker than that of cancellous bone), was slightly counteracted by the administration of diosmin, only at the lower dose.

**Table 3:** The effects of diosmin administered for 4 weeks on the mechanical properties of the femoral diaphysis and femoral neck in rats with STZ-induced T1D.

Parameter/Group	C	D	DIOS50	DIOS100	ANOVA	
Young’s modulus (MPa)	7093 ± 320	7458 ± 529	7552 ± 293	7125 ± 433	F_3,30_ = 0.352	*p* = 0.788
Yield point load (N)	97.9 ± 2.9	73.8 ± 2.9 ***	82.7 ± 3.2 ***,#	72.3 ± 1.3 ***	F_3,30_ = 18.600	*p* < 0.001
Displacement for yield point load (mm)	0.275 ± 0.004	0.247 ± 0.017	0.273 ± 0.010	0.254 ± 0.009	F_3,30_ = 1.722	*p* = 0.184
Energy for yield point load (mJ)	12.6 ± 0.5	8.9 ± 0.9 ***	10.9 ± 0.6 #	8.9 ± 0.3 ***	F_3,30_ = 8.106	*p* < 0.001
Stress for yield point load (MPa)	108.2 ± 3.5	98.3 ± 6.0	112.8 ± 5.6	99.3 ± 2.2	F_3,30_ = 2.318	*p* = 0.096
Maximum load (N)	139.2 ± 5.8	113.7 ± 6.4 **	116.7 ± 4.0 **	111.5 ± 3.5 ***	F_3,30_ = 6.623	*p* = 0.001
Displacement for maximum load (mm)	0.543 ± 0.034	0.497 ± 0.041	0.517 ± 0.031	0.530 ± 0.031	F_3,30_ = 0.327	*p* = 0.806
Energy for maximum load (mJ)	45.2 ± 4.9	34.0 ± 5.6	35.4 ± 3.1	34.5 ± 3.3	F_3,30_ = 1.544	*p* = 0.224
Stress for maximum load (MPa)	153.3 ± 4.4	150.0 ± 7.9	157.7 ± 2.3	153.1 ± 5.0	F_3,30_ = 0.390	*p* = 0.761
Fracture load (N)	139.1 ± 5.7	113.1 ± 6.1 ***	116.5 ± 4.0 **	109.8 ± 3.1 ***	F_3,30_ = 7.507	*p* < 0.001
Displacement for fracture load (mm)	0.548 ± 0.035	0.504 ± 0.045	0.519 ± 0.031	0.549 ± 0.041	F_3,30_ = 0.333	*p* = 0.801
Energy for fracture load (mJ)	45.8 ± 5.0	35.0 ± 6.2	35.7 ± 3.1	36.8 ± 4.6	F_3,30_ = 1.162	*p* = 0.341
Stress for fracture load (MPa)	153.1 ± 4.3	149.3 ± 7.7	157.5 ± 2.3	150.8 ± 4.9	F_3,30_ = 0.525	*p* = 0.669
Maximum load in femoral neck (N)	96.8 ± 4.2	84.8 ± 5.9	80.2 ± 5.1	80.5 ± 5.3	F_3,29_ = 2.220	*p* = 0.107

The results are presented as means ± the standard error of the mean (SEM; *n* = 8–9). C—healthy control rats (*n* = 9); D—diabetic control rats (*n* = 8); DIOS50—diabetic rats treated with diosmin at a dose of 50 mg/kg p.o. for 4 weeks (*n* = 9); DIOS100—diabetic rats treated with diosmin at a dose of 100 mg/kg p.o. for 4 weeks (*n* = 8). One-way analysis of variance (ANOVA) followed by Fisher’s LSD post hoc test were used for the evaluation of the significance of the results. Statistical significance in the post hoc test is indicated as follows: ** *p* < 0.01, *** *p* < 0.001 in comparison with the healthy control rats (C group); # *p* < 0.05 in comparison with the diabetic control rats (D group).

### 2.5. Effect of Diosmin on Histomorphometric Parameters of Femur in T1D Rats

In the diabetic control rats, the values of the bone volume/tissue volume ratio (BV/TV) and trabecular thickness (Tb.Th) in the distal femoral metaphysis were non-significantly decreased compared to the healthy control rats (Table 4; representative images—Figure S1). In the femoral diaphysis, diabetes reduced the values of the transverse cross-sectional area of the cortical bone (Ct.Ar) and the cross-sectional area of the whole diaphysis (Tt.Ar), with no significant effect on the cross-sectional area of the marrow cavity (Ma.Ar) and the cross-sectional area of the marrow cavity/total diaphysis area ratio (Ma.Ar/Tt.Ar; Table 4; representative images—Figure S2).

The administration of diosmin to diabetic rats had no significant effect on the histomorphometric parameters of the femoral metaphysis and diaphysis in relation to the diabetic control rats.

Summing up, diabetes-induced disorders of bone histomorphometric parameters were not counteracted by diosmin administration in rats.

## 3. Discussion

Chronic venous disease, which has an general prevalence ranging from 10% in adults under 30 years of age to nearly 80% in those over 70 years of age [35], is a serious public health problem. The pharmacological treatment options for chronic venous disease are rather limited and involve the use of flavonoids, including diosmin [36,37]. Diosmin is used in chronic venous disease in high doses, long-term [35].

Chronic venous disease and diabetes with its microvascular complications can occur simultaneously [36,37]. Therefore, the use of diosmin may be common in people with diabetes. Taking into account the effects of diabetes on the skeletal system and preclinical reports indicating the beneficial effect of diosmin on the skeletal system, it seemed reasonable to undertake studies on its effects on bone in experimental diabetes.

This study used an experimental model of T1D induced by a single injection of STZ in male rats. The rats exhibited high hyperglycemia (non-fasting glucose levels at the beginning of diosmin administration and in the following weeks reached more than 500 mg/dL) [29]. As previously reported [38], six weeks after the induction of diabetes, profound skeletal disorders were demonstrated in the control diabetic rats, consistent with our previous studies [39,40,41]. Decreased bone macrometric parameters, mass, density and mineral content were observed. The inhibition of bone formation and increased bone resorption led to the worsening of the mechanical properties of bone. In cancellous bone of the proximal tibial metaphysis, diabetes decreased both extrinsic (i.e., specimen size-dependent: load, energy) and intrinsic mechanical parameters (i.e., specimen size-independent: stress) at the yield, maximum load and fracture points, while in compact bone of the femoral diaphysis, it only affected extrinsic parameters (load, energy).

Given the pathophysiology of bone changes in diabetes, including the role of increased oxidative stress, we expected that diosmin might have beneficial effects on the skeletal system of diabetic rats. It has been proposed that diosmin, known for its antioxidant properties [30,42], may support antioxidant systems and show potential protective effects against osteoporosis [31]. The present study did not investigate parameters of oxidative stress in the skeletal system. However, a previous study in rats whose bones were examined in this study showed that diosmin reduced markers of oxidative stress in the lenses of diabetic rats [29].

In the present study, rats were administered diosmin at doses of 50 or 100 mg/kg p.o., which were approximately equivalent to the doses used in humans (500 or 1000 mg daily), accounting for the faster metabolism in rats [43]. Diosmin administration lasted four weeks; this period in rats corresponded to about 2.5 years in humans [44]. According to our previous studies, such a period of administration is sufficient to observe the effects of various plant-derived substances on the skeletal system of rats [39,40,45,46]. The administration of diosmin at doses of 50 and 100 mg/kg p.o. did not have a statistically significant effect on most of the skeletal parameters studied in rats with STZ-induced T1D. It was only shown that the administration of diosmin at the lower dose (50 mg/kg p.o.) resulted in a slight improvement in the bone mechanical parameters of diabetic rats. Under the influence of diosmin, Young’s modulus in the proximal metaphysis of the tibia, which consists mainly of cancellous bone, increased significantly compared to the diabetic controls. An increase in Young’s modulus, which is an intrinsic parameter, suggests that this improvement may have been due to an effect on collagen structure (for example, by inhibiting non-enzymatic glycation). It should be mentioned that cancellous bone is more susceptible to various factors than compact bone. However, diosmin at the lower dose also affected the mechanical properties of compact bone of the femoral diaphysis in diabetic rats, increasing the yield point load and energy; the improvement in compact bone mechanical properties was confirmed through PCA. Interestingly, no effect of diosmin on the skeletal system was observed when it was administered at the higher dose (100 mg/kg p.o.).

So far, no studies have been published on the effects of diosmin on the skeletal system under the conditions of hyperglycemia. In rats with glucocorticoid excess induced by dexamethasone administration, diosmin administered for four weeks inhibited bone resorption and increased bone formation. The antiresorptive effect was due to a decrease in the RANKL/OPG ratio, while the promotion of bone formation was due to the increased expression of Runx2, Wnt and osteocalcin. Most of these effects were observed after four weeks of oral administration of diosmin at a dose of 100 mg/kg, compared to a lower dose of 50 mg/kg [31]. Increased bone mass, improved trabecular bone microarchitecture and increased bone hardness were observed under the influence of diosmin (100 mg/kg p.o. for 45 days) in rats with chronic kidney disease induced by five-sixths nephrectomy, also due to increased bone formation and decreased bone resorption. Similar effects on bone microarchitecture were exerted by diosmetin, the aglycon of diosmin and its metabolite, in rats with chronic kidney disease [33]. In estrogen-deficient (ovariectomized) rats, the 12-week administration of diosmin (105 mg/kg p.o.) with hesperidin inhibited bone resorption and increased bone formation, leading to improvement in bone microarchitecture and strength [32]. Diosmin with hesperidin also promoted bone regeneration in vivo [32]. In vitro, diosmetin was found to inhibit sclerostin production and increase osteoblast differentiation [32]. Diosmin-loaded scaffolds promoted bone regeneration in mouse stem cell culture in vitro and in vivo in rats [34].

The slight beneficial effects of diosmin on the skeletal system in female rats may be related to its estrogen-like effects, since diosmin is classified as a phytoestrogen [33] and diosmetin acts as a selective estrogen receptor β agonist [32]. Taking into account the important role that estrogens play in male organisms [47], this mechanism of action of diosmin can also be considered in the present study conducted on male diabetic rats.

The effects of diosmin reported in earlier studies conducted on other models of bone loss [31,32,33] were much stronger than those demonstrated in this study. The inconsistency between the results obtained in this study and in other experimental models may be due to different experimental conditions; the T1D-induced changes in the skeletal system may be too profound to be counteracted by diosmin. The lack of a dose-dependent response to diosmin observed in this study may have been due to the possibly better antioxidant effect of the lower dose of diosmin in conditions of hyperglycemia. Such an effect was demonstrated for diosmin in vitro [48]. The fact that diosmin has very poor water solubility and limited bioavailability [20] may have contributed to its negligible effects on the rat skeletal system in the present study. On the other hand, diosmin is metabolized (hydrolyzed) into its aglycone diosmetin in the intestine; only diosmetin is found in the systemic circulation [32,33]. The skeletal effects of the administration of diosmetin resembled those of diosmin administration [33].

In conclusion, in this study, diosmin exerted a very weak beneficial effect on the mechanical properties of bone in rats under conditions of high hyperglycemia. This effect was too weak to infer the potential of diosmin to prevent the skeletal complications of diabetes, especially when taking into account the lack of a dose-dependent effect of diosmin. Although the results of the present study do not support the use of diosmin to counteract the skeletal complications of diabetes, the lack of an effect of diosmin on bone indicates that the long-term use of diosmin in chronic venous disease in patients with diabetes may be safe for the skeletal system.

## 4. Materials and Methods

### 4.1. Animals and In Vivo Experiments

The experiment was conducted on three-month-old male Wistar rats obtained from the Center of Experimental Medicine, Medical University of Silesia, Katowice. The in vivo study protocol was approved by the Local Ethics Committee, Katowice, Poland (approval numbers: 36/2015 and 114/2015). The conditions under which the rats were kept were in accordance with European Union guidelines (Directive 2010/63/EU). Artificial lighting operated on a cycle of 12 h of light and 12 h of darkness, and the light was turned on daily at 7:00 a.m. The rats were fed a standard laboratory diet (Labofeed B, Wytwórnia Pasz “Morawski”, Kcynia, Poland) ad libitum and had constant access to water throughout the experiment.

As previously reported [29], experimental T1D was induced by a single intraperitoneal (i.p.) injection of streptozotocin (STZ; Cayman Chemical, Ann Arbor, MI, USA) at a dose of 60 mg/kg as a solution in 0.1 M citrate buffer (in a volume of 1 mL/kg). Two weeks after the STZ injection, non-fasting glucose in blood collected from the tail vessels, obtained by cutting the tip of the tail, was measured using a MicroDot glucometer equipped with test strips (Cambridge Sensor USA, Plainfield, IL, USA). The upper limit of the glucose measurements was 525 mg/dL. Rats with blood glucose values above 200 mg/dL were considered diabetic.

The rats were assigned to the following groups:

– Group C—healthy (non-diabetic) control rats (*n* = 9; non-fasting glucose levels at the beginning of vehicle administration in the range of 97–145 mg/dL, and at the end in the range of 101–131 mg/dL).
– Group D—diabetic control rats (*n* = 8; non-fasting glucose levels at the beginning of vehicle administration ranging from 444 mg/dL to above the upper limit, and at the end ranging from 508 mg/dL to above the upper limit).
– Group DIOS50—diabetic rats receiving diosmin at a dose of 50 mg/kg (*n* = 9; non-fasting glucose levels at the beginning of diosmin administration ranging from 492 mg/dL to above the upper limit, and at the end ranging from 484 mg/dL to above the upper limit).
– Group DIOS100—diabetic rats receiving diosmin at a dose of 100 mg/kg (*n* = 8; non-fasting glucose levels at the beginning of diosmin administration ranging from 510 mg/dL to above the upper limit, and at the end ranging from 486 mg/dL to above the upper limit).

The details of the in vivo experiment were already described in our previous study [29]. This study shared control groups with our other study [38].

The administration of diosmin (Diosmin, Fluka Analytical, Sigma–Aldrich, St. Louis, MO, USA; catalog number: D3525) began two weeks after the STZ injection. Diosmin, suspended in water, was administered to rats once a day at a fixed time at a dose of 50 mg/kg or at a dose of 100 mg/kg by intragastric tube (per os—p.o.), at a volume of 1 mL/kg. The water suspensions of diosmin, in proportions of 50 or 100 mg/mL, were prepared every day before the administration. The control rats (group C and group D) were given water (the vehicle) at the same volume of 1 mL/kg p.o. Both diosmin and the vehicle were administered for four weeks.

The rats were weighed on the day of the STZ injection, two weeks later (before the start of diosmin or water administration) and after four weeks of diosmin or water administration. Non-fasting blood glucose levels were measured before the STZ injection, before the start of diosmin or water administration, and at the end of the experiment. Data on rat body mass changes and glycemia have already been reported in our previous article [29].

After four weeks of vehiculum or diosmin administration, rats were generally anesthetized by the intraperitoneal administration of a mixture of ketamine (Ketamina 10%, Biowet Puławy Sp. z o. o., Puławy, Poland) and xylazine (Xylapan, Vetoquinol Biowet, Gorzów Wlkp., Poland) and euthanized by cardiac exsanguination. The blood samples were centrifuged after clotting at room temperature. The collected serum was stored at a temperature of −80 °C until biochemical parameters were determined. Rat left femurs, left tibias and L4 vertebrae were isolated and weighed on an Adventurer Pro type AV264CM analytical balance (Ohaus Europe GmbH, Greifensee, Switzerland). The length and diameter of the left femur and tibia were determined using a VOREL 15240 digital caliper (Toya, Wrocław, Poland). The bones were wrapped in gauze soaked in 0.9% NaCl solution and stored at a temperature below −20 °C until mechanical tests were performed on the thawed bones.

### 4.2. Measurements of Serum Biochemical Parameters

Serum markers of bone turnover—osteocalcin (a marker of bone formation) and C-terminal telopeptide fragments of type I collagen (CTX-I; a marker of bone resorption)—were measured by ELISA using Rat-MID Osteocalcin EIA and RatLaps (CTX-I) EIA kits (Immunodiagnostic Systems Ltd., Boldon, Tyne and Wear, UK), respectively. Measurements were performed using a Tecan Infinite M200 Pro plate reader with Magellan 7.2 software (Tecan Austria, Grödig, Austria). Serum concentrations of calcium and inorganic phosphorus were determined with Pointe Scientific kits (Canton, MI, USA). A Tecan Infinite M200 Pro plate reader with Magellan 7.2 software (Tecan Austria, Grödig, Austria) was used for all measurements.

### 4.3. Bone Composition Measurements

To remove water, freeze-drying was carried out at −53 °C and 0.03 mBa for ten days in a Labconco FreeZone 6 freeze-dryer (Labconco, Kansas City, MO, USA). The freeze-dried bones were mineralized at 640 °C for 48 h in a L9/11/C6 muffle furnace (Nabertherm, Lilienthal, Germany). The mass of minerals, water and organic substances in the bones was determined, and the bone content of minerals, water and organic substances was calculated as the ratio to bone mass, as previously described [38]. In the next stage of the study, the mineralized bones were dissolved in 6 M HCl and then diluted in deionized water for the spectrophotometric determination of calcium and phosphorus content in the bone minerals. Calcium and phosphorus contents were determined spectrophotometrically using an automatic biochemical analyzer (BS-240, Mindray, Shenzhen, China) and Pointe Scientific (Canton, MI, USA) kits.

The bone densities of the left tibias (after the removal of the proximal epiphysis), left femurs and L4 vertebrae were assessed according to Archimedes’ principle [49]. Measurements were made using an Adventurer Pro AV264CM analytical balance with a density determination kit (OHAUS Europe GmbH, Greifensee, Switzerland). Bone mineral density was calculated as the ratio of bone mineral mass to bone volume.

### 4.4. Bone Mechanical Properties Measurements

Bone mechanical properties were determined using an Instron 3342 500N (Instron, Norwood, MA, USA) apparatus, as previously described [41,46,50,51]. The results were analyzed using Bluehill 2 software version 2.14 (Instron, Norwood, MA, USA). The mechanical properties of the left femoral diaphysis and proximal tibial metaphysis were studied using three-point bending tests. The mechanical properties of the right femoral neck were determined using a compression test. The tests were performed with a displacement rate of 0.01 mm/s and a sampling frequency of 100 Hz.

In the bending tests, the load was applied perpendicularly to the femoral diaphysis at the bone mid-length, with the support points 16 mm apart, or to the tibia deprived of the proximal epiphysis, 3 mm from the edge of the proximal tibial metaphysis. During the tests, the following parameters were determined: Young’s modulus and the load, displacement, energy and stress for the yield point (0.05% offset), maximum load point and fracture point. The moment of inertia required to determine Young’s modulus and the stress values was estimated assuming that the femoral diaphysis was an elliptical pipe and the tibial metaphysis was a circular beam, as previously described [41,46].

To assess the strength of the femoral neck, the proximal part of the right femur (cut in the middle of the bone’s length) was fixed to a polymethyl methacrylate plate. A load was applied to the femoral head along the longitudinal axis of the bone. The maximum load causing a femoral neck fracture was determined.

### 4.5. Bone Histomorphometric Measurements

Histological preparations were prepared from the right femur as previously described [41,46]. Histomorphometric measurements were performed using an Axio Imager.A1 microscope (Carl Zeiss, Göttingen, Germany) coupled to an Olympus DP71 camera (Olympus, Tokyo, Japan) and OsteoMeasure XP v1.3.0.1 software (OsteoMetrics, Decatur, GA, USA). Histomorphometric data were presented according to the standardized nomenclature of the American Society for Bone and Mineral Research (ASBMR) [52].

In non-decalcified, unstained cross-sectional slides of the femoral diaphysis, Ct.Ar, Ma.Ar, Tt.Ar and Ma.Ar/Tt.Ar were determined.

In decalcified and hematoxylin and eosin-stained longitudinal sections of the distal femur, BV/TV, Tb.Th, Tb.Sp and Tb.N in the femoral metaphysis were measured.

### 4.6. Statistical Analysis

Results are presented as means ± standard error of the mean (SEM). The results were statistically analyzed using the Statistica 13.3 program (Tibco Software Inc., Palo Alto, CA, USA). One-way analysis of variance (ANOVA) followed by Fisher’s least significant difference (LSD) post hoc test was performed to assess the statistical significance of the results. The results of the groups of rats with diabetes were compared with those of the healthy control rats, and the results of the diabetic rats treated with diosmin were compared with the control rats with diabetes. The results were considered statistically significant if *p* < 0.05.

Moreover, principal component analysis (PCA) was performed on the results concerning bone mechanical properties with PAST 5.0.1 software (Natural History Museum, University of Oslo, Norway), based on the correlation matrix [53]. The statistical significance of the PCA results was evaluated using multivariate analysis of variance (MANOVA) followed by Fisher’s LSD post hoc test (Statistica 13.3). The following groups of parameters were subjected to PCA:

- The mechanical properties of cancellous bone (the proximal tibial metaphysis): yield point load in the tibial metaphysis; displacement, energy and stress for yield point load in the tibial metaphysis; maximum load in the tibial metaphysis; displacement, energy and stress for maximum load in the tibial metaphysis; fracture load in the tibial metaphysis; displacement, energy and stress for fracture load in the tibial metaphysis; Young’s modulus in the tibial metaphysis.
- The mechanical properties of compact bone (the femoral diaphysis): yield point load in the femoral diaphysis; displacement, energy and stress for yield point load in the femoral diaphysis; maximum load in the femoral diaphysis; displacement, energy and stress for maximum load in the femoral diaphysis; fracture load in the femoral diaphysis; displacement, energy and stress for fracture load in the femoral diaphysis; Young’s modulus in the femoral diaphysis.

## Figures and Tables

**Table 1:** The effects of diosmin administered for 4 weeks on the bone macrometric parameters, mass, density, mineral density and mineralization in the femur of rats with STZ-induced T1D.

Parameter/Group	C	D	DIOS50	DIOS100	ANOVA	
Bone length (mm)	36.40 ± 0.34	34.28 ± 0.31 ***	34.68 ± 0.23 ***	34.67 ± 0.24 ***	F_3,30_ = 11.241	*p* < 0.001
Bone diameter (mm)	3.60 ± 0.05	3.33 ± 0.09 **	3.35 ± 0.05 **	3.37 ± 0.07 *	F_3,30_ = 3.978	*p* = 0.017
Bone mass (g)	0.855 ± 0.022	0.702 ± 0.024 ***	0.713 ± 0.014 ***	0.697 ± 0.020 ***	F_3,30_ = 15.027	*p* < 0.001
Bone density (g/cm^3^)	1.626 ± 0.005	1.551 ± 0.012 ***	1.576 ± 0.013 **	1.562 ± 0.010 ***	F_3,30_ = 10.705	*p* < 0.001
Bone mineral density (g/cm^3^)	0.753 ± 0.006	0.695 ± 0.010 ***	0.712 ± 0.010 **	0.695 ± 0.011 ***	F_3,30_ = 8.374	*p* < 0.001
Bone mineral mass (g)	0.396 ± 0.009	0.314 ± 0.010 ***	0.322 ± 0.006 ***	0.310 ± 0.009 ***	F_3,30_ = 23.629	*p* < 0.001
Bone water mass (g)	0.259 ± 0.007	0.218 ± 0.010 **	0.221 ± 0.007 **	0.218 ± 0.009 **	F_3,30_ = 5.919	*p* = 0.003
Bone organic substances mass (g)	0.201 ± 0.006	0.169 ± 0.007 ***	0.170 ± 0.003 ***	0.168 ± 0.004 ***	F_3,30_ = 9.603	*p* < 0.001
Bone mineral mass/bone mass ratio	0.463 ± 0.003	0.448 ± 0.005 *	0.452 ± 0.004	0.445 ± 0.005 **	F_3,30_ = 3.696	*p* = 0.022
Bone water mass/bone mass ratio	0.302 ± 0.003	0.310 ± 0.006	0.309 ± 0.006	0.313 ± 0.005	F_3,30_ = 0.778	*p* = 0.515
Bone organic substances mass/bone mass ratio	0.234 ± 0.002	0.241 ± 0.006	0.239 ± 0.004	0.242 ± 0.003	F_3,30_ = 0.785	*p* = 0.512
Calcium content (g/g of bone mineral)	0.422 ± 0.003	0.419 ± 0.003	0.421 ± 0.005	0.418 ± 0.002	F_3,30_ = 0.331	*p* = 0.803
Phosphorus content (g/g of bone mineral)	0.171 ± 0.001	0.167 ± 0.001 *	0.167 ± 0.001 **	0.167 ± 0.001 *	F_3,30_ = 3.549	*p* = 0.026

The results are presented as means ± the standard error of the mean (SEM; *n* = 8–9). C—healthy control rats (*n* = 9); D—diabetic control rats (*n* = 8); DIOS50—diabetic rats treated with diosmin at a dose of 50 mg/kg p.o. for 4 weeks (*n* = 9); DIOS100—diabetic rats treated with diosmin at a dose of 100 mg/kg p.o. for 4 weeks (*n* = 8). One-way analysis of variance (ANOVA) followed by Fisher’s LSD post hoc test were used for the evaluation of the significance of the results. Statistical significance in the post hoc test is indicated as follows: * *p* < 0.05, ** *p* < 0.01, *** *p* < 0.001 in comparison with the healthy control rats (C group).

**Table 2:** The effects of diosmin administered for 4 weeks on the mechanical properties of the proximal tibial metaphysis in rats with STZ-induced T1D.

Parameter/Group	C	D	DIOS50	DIOS100	ANOVA	
Displacement for yield point load (mm)	0.285 ± 0.062	0.217 ± 0.025	0.134 ± 0.016 **	0.183 ± 0.017 *	F_3,29_ = 3.401	*p* = 0.031
Maximum load (N)	65.0 ± 3.2	31.9 ± 2.3 ***	32.8 ± 2.6 ***	29.6 ± 1.2 ***	F_3,29_ = 46.270	*p* < 0.001
Displacement for maximum load (mm)	0.903 ± 0.060	0.531 ± 0.078	0.764 ± 0.220	0.623 ± 0.072	F_3,29_ = 1.434	*p* = 0.253
Energy for maximum load (mJ)	37.2 ± 3.0	10.7 ± 1.9 ***	18.2 ± 5.7 ***	12.4 ± 1.4 ***	F_3,29_ = 10.843	*p* < 0.001
Stress for maximum load (MPa)	46.0 ± 2.7	25.6 ± 2.1 ***	27.6 ± 2.2 ***	26.2 ± 1.4 ***	F_3,29_ = 19.847	*p* < 0.001
Fracture load (N)	48.7 ± 3.3	24.1 ± 2.5 ***	25.0 ± 2.7 ***	24.5 ± 1.8 ***	F_3,29_ = 20.205	*p* < 0.001
Displacement for fracture load (mm)	1.396 ± 0.065	0.911 ± 0.070 *	1.063 ± 0.199	0.945 ± 0.087 *	F_3,29_ = 2.994	*p* = 0.047
Energy for fracture load (mJ)	65.0 ± 3.7	21.4 ± 2.4 ***	26.5 ± 5.6 ***	21.1 ± 2.5 ***	F_3,29_ = 28.331	*p* < 0.001
Stress for fracture load (MPa)	34.8 ± 3.2	19.3 ± 2.1 ***	21.1 ± 2.5 ***	21.5 ± 1.5 ***	F_3,29_ = 8.581	*p* < 0.001

The results are presented as means ± the standard error of the mean (SEM; *n* = 8–9). C—healthy control rats (*n* = 9); D—diabetic control rats (*n* = 8); DIOS50—diabetic rats treated with diosmin at a dose of 50 mg/kg p.o. for 4 weeks (*n* = 9); DIOS100—diabetic rats treated with diosmin at a dose of 100 mg/kg p.o. for 4 weeks (*n* = 8). One-way analysis of variance (ANOVA) followed by Fisher’s LSD post hoc test were used for the evaluation of the significance of the results. Statistical significance in the post hoc test is indicated as follows: * *p* < 0.05, ** *p* < 0.01, *** *p* < 0.001 in comparison with the healthy control rats (C group).

**Table 4:** The effects of diosmin administered for 4 weeks on histomorphometric parameters of the femoral metaphysis and diaphysis in rats with STZ-induced T1D.

Parameter/Group		C	D	DIOS50	DIOS100	ANOVA	
Femoral metaphysis	BV/TV (%)	33.05 ± 2.21	27.89 ± 4.55	28.54 ± 1.68	24.27 ± 0.78	F_3,18_ = 2.628	*p* = 0.082
	Tb.Th (μm)	47.34 ± 2.85	38.70 ± 5.40	36.76 ± 3.0 *	34.53 ± 1.40 **	F_3,18_ = 3.170	*p* = 0.050
	Tb.Sp (μm)	96.27 ± 4.58	102.02 ± 9.80	92.24 ± 6.27	109.02 ± 7.95	F_3,18_ = 1.161	*p* = 0.352
	Tb.N (1/mm)	6.98 ± 0.17	7.13 ± 0.25	7.09 ± 0.21	7.11 ± 0.47	F_3,18_ = 0.042	*p* = 0.988
Femoral diaphysis	Tt.Ar (mm^2^)	9.543 ± 0.235	8.617 ± 0.272 *	8.547 ± 0.324 *	8.374 ± 0.192 **	F_3,26_ = 4.538	*p* = 0.011
	Ct.Ar (mm^2^)	5.927 ± 0.193	5.224 ± 0.116 ***	5.275 ± 0.101 **	5.013 ± 0.061 ***	F_3,26_ = 9.630	*p* < 0.001
	Ma.Ar (mm^2^)	3.616 ± 0.199	3.392 ± 0.175	3.272 ± 0.231	3.361 ± 0.147	F_3,26_ = 0.606	*p* = 0.617
	Ma.Ar/Tt.Ar (%)	37.8 ± 1.6	39.2 ± 0.9	38.0 ± 1.4	40.0 ± 0.9	F_3,26_ = 0.698	*p* = 0.562

The results are presented as means ± the standard error of the mean (SEM; *n* = 4–7 for the metaphysis; *n* = 6–8 for the diaphysis). C—healthy control rats (*n* = 9); D—diabetic control rats (*n* = 8); DIOS50—diabetic rats treated with diosmin at a dose of 50 mg/kg p.o. for 4 weeks (*n* = 9); DIOS100—diabetic rats treated with diosmin at a dose of 100 mg/kg p.o. for 4 weeks (*n* = 8). BV/TV—bone volume/tissue volume ratio; Tb.Th—trabecular thickness; Tb.Sp—trabecular separation; Tb.N—trabecular number; Tt.Ar—the transverse cross-sectional area of the whole diaphysis; Ct.Ar—the transverse cross-sectional area of the cortical bone; Ma.Ar—the transverse cross-sectional area of the marrow cavity; Ma.Ar/Tt.Ar—the transverse cross-sectional area of the marrow cavity/total diaphysis area ratio. One-way analysis of variance (ANOVA) followed by Fisher’s LSD post hoc test were used for the evaluation of the significance of the results. Statistical significance in the post hoc test is indicated as follows: * *p* < 0.05, ** *p* < 0.01, *** *p* < 0.001 in comparison with the healthy control rats (C group).

## Data Availability

Data are contained within the article.

## Supplementary Materials

The following supporting information can be downloaded at: https://www.mdpi.com/article/10.3390/ph18050715/s1, Table S1: The effects of diosmin administered for 4 weeks on bone macrometric parameters, mass, density, mineral density and mineralization in the tibia deprived of the proximal epiphysis in rats with STZ-induced T1D; Table S2. The effects of diosmin administered for 4 weeks on bone mass, density, mineral density and mineralization in the L4 vertebra in rats with STZ-induced T1D; Figure S1. Representative images of the decalcified, hematoxylin and eosin-stained longitudinal sections of the distal femoral metaphysis; Figure S2. Representative images of the unstained transverse cross-sections of the femoral diaphysis.

## Abbreviations

The following abbreviations are used in this manuscript:

ANOVA	analysis of variance
BV/TV	bone volume/tissue volume ratio
Ct.Ar	transverse cross-sectional area of the cortical bone
CTX-I	C-terminal telopeptide fragments of type I collagen
LSD	least significant difference
Ma.Ar	transverse cross-sectional area of the marrow cavity
Ma.Ar/Tt.Ar	transverse cross-sectional area of the marrow cavity/total diaphysis area ratio
MANOVA	multivariate analysis of variance
PCA	principal component analysis
p.o.	per os
STZ	streptozotocin
T1D	type 1 diabetes
Tb.N	trabecular number
Tb.Sp	trabecular separation
Tb.Th	trabecular thickness
Tt.Ar	transverse cross-sectional area of the whole diaphysis
