# Supplementary material for: Diosmin Administration Slightly Counteracted the Changes in Bone Mechanical Properties Induced by Experimental Type 1 Diabetes in Rats
Source: Pharmaceuticals (Basel). 2025 May 13;18(5):715. doi: 10.3390/ph18050715 (PMC12114974; doi:10.3390/ph18050715)
Supplement: Supplementary file 1 [file pharmaceuticals-18-00715-s001.zip › pharmaceuticals-3592950-supplementary.pdf]

pharmaceuticals

MDPI

Supplementary materials

Table S1. The effects of diosmin administered for 4 weeks on bone macrometric parameters, mass, density, mineral density, and mineralization in the tibia deprived of the proximal epiphysis in rats with STZ-induced T1D.

Parameter/Group	C	D	DIOS50	DIOS100	ANOVA	
Bone length[&] (mm)	40.17 ± 0.34	38.46 ± 0.37***	38.45 ± 0.26***	38.69 ± 0.31**	$F_{3,30} = 6.955$	$p = 0.001$
Bone diameter[&] (mm)	2.88 ± 0.05	2.74 ± 0.05*	2.71 ± 0.04 *	2.71 ± 0.04*	$F_{3,30} = 3.308$	$p = 0.033$
Bone mass (g)	0.535 ± 0.012	0.438 ± 0.017***	0.436 ± 0.009***	0.428 ± 0.011***	$F_{3,29} = 16.758$	$p < 0.001$
Bone density (g/cm^3)	1.636 ± 0.007	1.586 ± 0.009**	1.592 ± 0.012**	1.587 ± 0.011**	$F_{3,30} = 6.219$	$p = 0.002$
Bone mineral density (g/cm^3)	0.776 ± 0.008	0.723 ± 0.008***	0.736 ± 0.007***	0.736 ± 0.007***	$F_{3,29} = 9.690$	$p < 0.001$
Bone mineral mass (g)	0.253 ± 0.005	0.200 ± 0.007***	0.202 ± 0.005***	0.199 ± 0.005***	$F_{3,29} = 22.761$	$p < 0.001$
Bone water mass (g)	0.148 ± 0.004	0.129 ± 0.008*	0.125 ± 0.005**	0.118 ± 0.004***	$F_{3,29} = 6.168$	$p = 0.002$
Bone organic substances mass (g)	0.133 ± 0.004	0.109 ± 0.005***	0.109 ± 0.003***	0.112 ± 0.003***	$F_{3,29} = 9.995$	$p < 0.001$
Bone mineral mass/bone mass ratio	0.474 ± 0.003	0.456 ± 0.003***	0.464 ± 0.004*	0.464 ± 0.003*	$F_{3,29} = 4.843$	$p = 0.007$
Bone water mass/bone mass ratio	0.276 ± 0.005	0.294 ± 0.009	0.286 ± 0.009	0.275 ± 0.005	$F_{3,29} = 1.574$	$p = 0.217$
Bone organic substances mass/bone mass ratio	0.249 ± 0.003	0.250 ± 0.008	0.250 ± 0.007	0.261 ± 0.004	$F_{3,29} = 0.992$	$p = 0.410$
Calcium content (g/g of bone mineral)	0.423 ± 0.003	0.429 ± 0.003	0.427 ± 0.004	0.433 ± 0.003	$F_{3,30} = 1.653$	$p = 0.198$
Phosphorus content (g/g of bone mineral)	0.172 ± 0.002	0.170 ± 0.002	0.169 ± 0.001	0.171 ± 0.002	$F_{3,30} = 0.522$	$p = 0.670$

The results are presented as means ± standard error of the mean (SEM; n=8-9). C—healthy control rats (n=9); D—diabetic control rats (n=8); DIOS50—diabetic rats treated with diosmin at a dose of 50 mg/kg p.o. for 4 weeks (n=9); DIOS100—diabetic rats treated with diosmin at a dose of 100 mg/kg p.o. for 4 weeks (n=8). [&]—whole tibia. One-way analysis of variance (ANOVA) followed by Fisher's LSD *post hoc* test was used for evaluation of the significance of the results. Statistical significance in *post hoc* test: * $p < 0.05$, ** $p < 0.01$, *** $p < 0.001$—in comparison with the healthy control rats (C group).

Table S2. The effects of diosmin administered for 4 weeks on bone mass, density, mineral density, and mineralization in the L4 vertebra in rats with STZ-induced T1D.

Parameter/Group	C	D	DIOS50	DIOS100	One-way ANOVA	
Bone mass (g)	0.208 ± 0.010	0.155 ± 0.005***	0.159 ± 0.007***	0.145 ± 0.010***	$F_{3,30} = 11.383$	$p < 0.001$
Bone density (g/cm^3)	1.559 ± 0.013	1.472 ± 0.015	1.545 ± 0.068	1.503 ± 0.013	$F_{3,30} = 1.070$	$p = 0.377$
Bone mineral density (g/cm^3)	0.703 ± 0.009	0.651 ± 0.019	0.676 ± 0.036	0.662 ± 0.011	$F_{3,30} = 0.991$	$p = 0.410$
Bone mineral mass (g)	0.094 ± 0.004	0.069 ± 0.003***	0.069 ± 0.003***	0.064 ± 0.005***	$F_{3,30} = 11.691$	$p < 0.001$
Bone water mass (g)	0.062 ± 0.004	0.048 ± 0.002**	0.051 ± 0.003**	0.045 ± 0.003***	$F_{3,30} = 6.817$	$p = 0.001$
Bone organic substances mass (g)	0.052 ± 0.002	0.038 ± 0.002***	0.039 ± 0.002***	0.036 ± 0.003***	$F_{3,30} = 11.390$	$p < 0.001$
Bone mineral mass/bone mass ratio	0.451 ± 0.004	0.442 ± 0.010	0.436 ± 0.006	0.441 ± 0.007	$F_{3,30} = 0.843$	$p = 0.481$
Bone water mass/bone mass ratio	0.300 ± 0.006	0.310 ± 0.013	0.318 ± 0.008	0.309 ± 0.009	$F_{3,30} = 0.727$	$p = 0.544$
Bone organic substances mass/bone mass ratio	0.249 ± 0.002	0.248 ± 0.004	0.245 ± 0.002	0.250 ± 0.003	$F_{3,30} = 0.460$	$p = 0.712$
Calcium content (g/g of bone mineral)	0.436 ± 0.010	0.444 ± 0.008	0.440 ± 0.008	0.452 ± 0.009	$F_{3,30} = 0.539$	$p = 0.659$
Phosphorus content (g/g of bone mineral)	0.171 ± 0.001	0.169 ± 0.002	0.170 ± 0.002	0.169 ± 0.001	$F_{3,30} = 0.219$	$p = 0.882$

The results are presented as means ± standard error of the mean (SEM; n=8-9). C—healthy control rats (n=9); D—diabetic control rats (n=8); DIOS50—diabetic rats treated with diosmin at a dose of 50 mg/kg p.o. for 4 weeks (n=9); DIOS100—diabetic rats treated with diosmin at a dose of 100 mg/kg p.o. for 4 weeks (n=8). One-way analysis of variance (ANOVA) followed by Fisher's LSD *post hoc* test was used for evaluation of the significance of the results. Statistical significance in *post hoc* test: ** $p < 0.01$, *** $p < 0.001$—in comparison with the healthy control rats (C group).

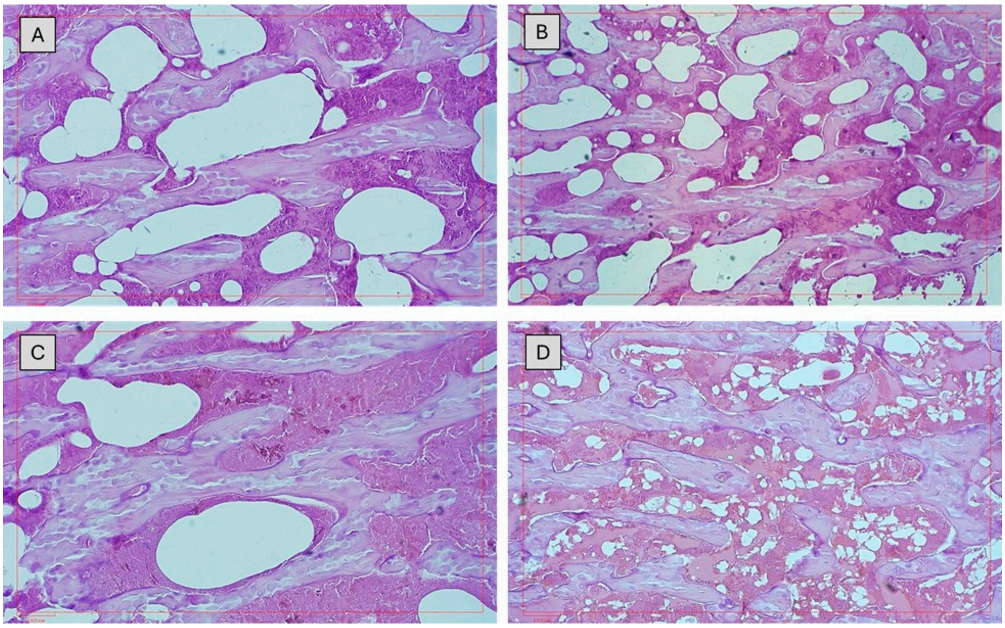

Figure S1. Representative images of the decalcified, hematoxylin and eosin-stained longitudinal sections of the distal femoral metaphysis. A—healthy control rats; B—diabetic control rats; C—diabetic rats treated with diosmin at a dose of 50 mg/kg p.o. for 4 weeks; D—diabetic rats treated with diosmin at a dose of 100 mg/kg p.o. for 4 weeks.

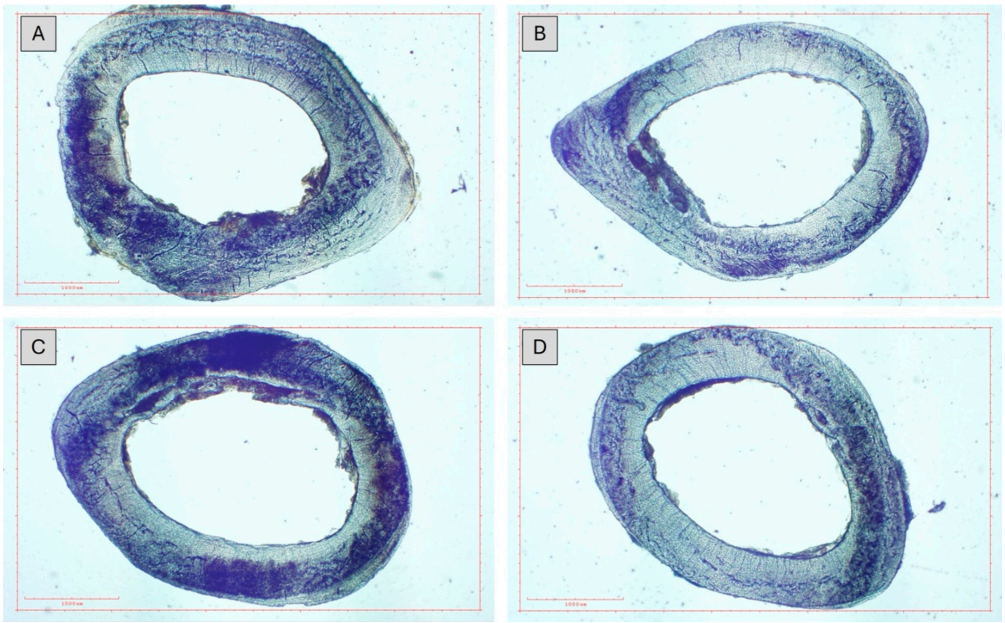

Figure S2. Representative images of the unstained transverse cross-sections of the femoral diaphysis. A—healthy control rats; B—diabetic control rats; C—diabetic rats treated with diosmin at a dose of 50 mg/kg p.o. for 4 weeks; D—diabetic rats treated with diosmin at a dose of 100 mg/kg p.o. for 4 weeks.
